# Two hours method for RNA and DNA co-extraction from blood of coronary artery disease patients: Fast, simple and economical technique

**DOI:** 10.12669/pjms.38.7.5509

**Published:** 2022

**Authors:** Abdullahi Dandare, Muhammad Rafiq, Afrose Liaquat, Muhammad Jawad Khan

**Affiliations:** 1 Abdullahi Dandare, Department of Biochemistry, Usmanu Danfodiyo University Sokoto, Nigeria. Department of Biosciences, COMSATS University Islamabad, 45550, Pakistan; 2 Muhammad Rafiq, Department of Biosciences, COMSATS University Islamabad, 45550, Pakistan, Department of Biochemistry, Shifa College of Medicine, Shifa Tameer-E-Millat University, Islamabad, 45550, Pakistan; 3 Afrose Liaquat, Department of Biochemistry, Shifa College of Medicine, Shifa Tameer-E-Millat University, Islamabad, 45550, Pakistan; 4 Muhammad Jawad Khan, Department of Biosciences, COMSATS University Islamabad, 45550, Pakistan

**Keywords:** DNA extraction, RNA extraction, Cost-effective, Co-extraction, Coronary artery disease

## Abstract

**Objectives:**

Extraction of DNA and RNA is the first step in genomics and transcriptomics studies. Phenol-chloroform method for DNA extraction has been the widely used method. However, this method is relatively expensive and time-consuming. The objective of the present study was to validate a cost and time-effective protocol that will reduce the burden of molecular biology-based research and make a difference in laboratories with limited resources.

**Methods:**

A comparative study was conducted at Syed Qamer Alam Research Laboratory, Shifa College of Medicine; from February, 2021 to August, 2021. TRIzol™ method was used to extract RNA from blood samples of coronary artery disease patients and remnant was used to extract DNA. The quantity, purity and integrity of the extracted DNA by both methods (TRIzol and phenol-chloroform) was examined. PCR product amplification was performed with *thrombomodulin (THBD)* gene to validate the characteristic of the extracted DNA and its efficiency for downstream experiments.

**Results:**

The DNA yield in the TRIzol™ method was three-fold higher than phenol chloroform method. Both methods showed intact genomic DNA on the agarose gel, and extracted DNA was efficient for PCR amplification.

**Conclusion:**

The TRIzol™ method for RNA and DNA co-extraction is fast, simple and economical technique. So, it can be adopted for routine molecular biology analyses in limited resources setup.

## INTRODUCTION

Nucleic acid extraction from biological samples is an integral part of many diagnostic and research procedures.^1^,^2^ It is the first step in many of the existing techniques used in the field of biochemistry and molecular biology.^3^ Different strategies have been employed in nucleic acid extraction to achieve high quality and quantity of the extracted DNA and RNA.^4^ The existing extraction and purification procedures for DNA and RNA vary from traditional or manual protocols to highly sophisticated techniques. Therefore, there is a wide range of options available for the nucleic acid extraction.^5^

Few of the determinant factors for the selection of appropriate procedure for nucleic acid extraction; include the required molecular weight of the target molecule, purity and quantity. In addition, cost-effectiveness, time efficiency, collection and storage requirements of the biological specimens are also very important factors to consider.^5^,^6^ In common practice, DNA and RNA molecules are extracted separately from the same sample which is time-consuming and a costly process; especially when dealing with a large number of samples. RNA extraction using TRIzol™ reagent is not a new protocol but the use of the left-over material, after the RNA has been separated, to extract DNA is a neglected practice. This will reduce the capital expenses and the time required to extract DNA molecules. It is not economically wise to use TRIzol™ reagent for RNA extraction and discard the other portion that contains DNA. The present protocol was designed to extract RNA from blood samples of coronary artery disease (CAD) patients using the conventional TRIzol method and process the leftover specimen to obtain the DNA that can be used in downstream experiments.

## METHODS

A comparative study was conducted at Syed Qamer Alam research laboratory, Shifa College of Medicine; from February, 2021 to August, 2021. The study was reviewed and approved by institutional review and ethics board of Shifa Tameer-e-Millat University (IRB # 016–506-2019).

### Collection and Transportation of Blood Samples

The description of sample collection has been previously reported.^7^ Briefly, 3ml of blood samples from CAD patients was dispensed in EDTA (Ethylenediamine tetraacetic acid) tubes and transported to the laboratory within one hour of collection and processed immediately.^7^

### RNA and DNA Co-Extraction Procedure:

### Step-1: Homogenization:

Total volume of 300 ul of blood sample was dispensed into a microtube (Eppendorf, Hamburg Germany) and mixed with 250 ul of DEPC (Diethylpyrocarbonate) water. Then 750 ul of TRIzol™ reagent (Thermo Fisher Scientific, Waltham MA USA) was added and mixed by repetitive pipetting to lyse the cells. The mixture was incubated at room temperature (25°C) for five minutes to allow the complete dissociation of nucleoprotein complexes. This was followed by the addition of 200 ul of chloroform (Sigma Aldrich, St. Louis MO USA) to promote phase separation. Vigorous shaking for 10-15 seconds was done till chocolate color was obtained. The mixture was re-incubated at room temperature for 10 minutes.

### Step- 2: Aqueous Phase Separation

The incubated mixture was centrifuged at 12500 rpm at 4°C for 15 minutes. This led to separation of upper transparent aqueous phase which contains RNA, followed by white-colored interphase containing DNA and the lowermost organic phase which contains proteins and other macromolecules. The aqueous phase was carefully pipetted out, without any shake to disturb the interphase and dispensed it into another microtube for further RNA processing. The remaining supernatant (interphase) was carefully transferred into separate microtube to obtain DNA. To achieve the extraction of both molecules in two hours, the DNA and RNA were processed concurrently. The next incubation time (30 minutes) for DNA processing after the addition of absolute ethanol is more than enough to process RNA molecule. While waiting for the RNA to dry before re-suspension into DEPC water, one can proceed with the procedure of DNA extraction.

### Step-3: Precipitation of RNA

500 ul of chilled isopropanol (Sigma Aldrich, St. Louis MO USA) was dispensed into the microtube containing aqueous phase. After gentle mixing through inversion, a suspension of fine jelly-like thread can be observed in the solution. The mixture was then incubated for 10 minutes at room temperature for precipitation of RNA (better yield can be obtained if incubation is made at -20°C). The mixture was centrifuged at 13000 rpm for 10 minutes at 4°C, and at least 3/4 of the supernatant was discarded**.** The remaining RNA pellet was washed by adding 1 ml of DEPC treated ethanol (Sigma Aldrich, St. Louis MO USA) for purification. It was then centrifuged again at 7500 rpm at 4°C for 5 minutes to clean the RNA pellet. The supernatant was discarded, and RNA pellet was air-dried at room temperature for approximately 10 minutes. RNAse free water (DEPC) was added to dissolve the dried RNA pellets and stored at -80°C.

### Step-4: DNA Precipitation

Absolute ethanol (750 ul) was added to the interphase and mixed gently by inversion. After 30 minutes incubation at room temperature, the mixture was centrifuged at 5000 rpm for 5 minutes at 4°C and the supernatant was discarded. The pellet was washed twice with 750 ul of 10% ethanol and 70% ethanol with 10 minutes incubation at the end of each washing. Final centrifugation was conducted at 5000 rpm, at 4°C for five minutes. The supernatant was discarded, and DNA pellet was left behind. The tube was air dried at room temperature for 7-10 minutes. The dried DNA pellet was dissolved in 30 ul of 8 mM NaOH and stored at -20°C.^8^

### Phenol-Chloroform Method

DNA was also extracted with phenol-chloroform method, described by Kochl et al.^9^ The extracted DNA was immediately evaluated for quantity, purity and integrity. Results obtained were compared with the present TRIzol™ optimized method.

### DNA Purity and Quantity Assessment

The purity and quantity of DNA were spectrophotometrically assessed using Thermo Scientific Multiskan GO micro-plate spectrophotometer. Absorbance was measured at wavelengths of 320, 280 and 260 nm. Samples were analyzed in duplicate and the average of the absorbance was used to ascertain the yield and purity of the DNA. The absorbance ratios of 260 and 280 nm (A_260_/A_280_) were used to assess the purity of the extracted DNA. The absorbance ratio of 1.8-2.0 was accepted as good DNA purity.^10^,^11^ Proteins contamination may result in the absorbance ratio of less than 1.8, while the RNA contamination makes the ratio above 2.0.^11^,^12^

### DNA Integrity Assessment

The integrity of the extracted DNA, was assessed immediately and after two months of extraction through gel electrophoresis system. The DNA samples were analysed on 0.8% agarose gel and viewed under UV radiation in gel documentation system (ethidium bromide used as fluorescent tag).^13^ Computer digital image analysis was done to evaluate the agarose gel band density of the extracted DNA. Peaks were automatically generated by the ImageJ software (Fig.1), and analyzed to obtained relative percentage of each band in the agarose gel.^14^

**Fig.1a:**
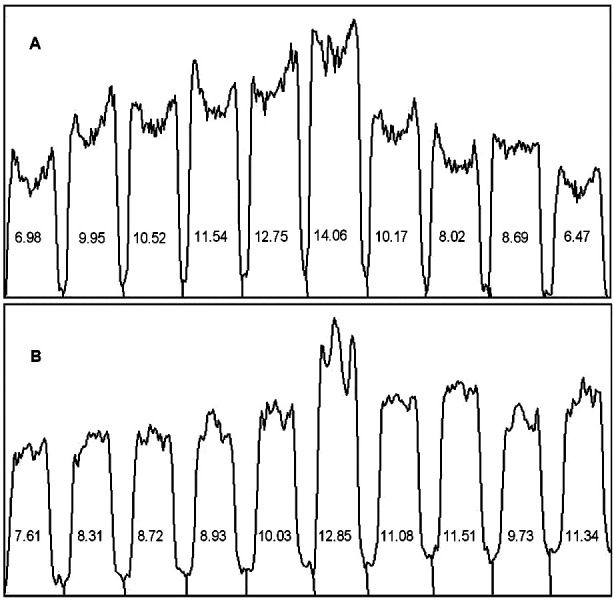
Image J generated peaks of the corresponding extracted DNA. These peaks represent DNA band densities of samples 1-10, and within peaks, are the respective relative percentage of each DNA band on the agarose gel. A: phenol chloroform method, B: modified TRIzol™ method.

**Fig.1b:**
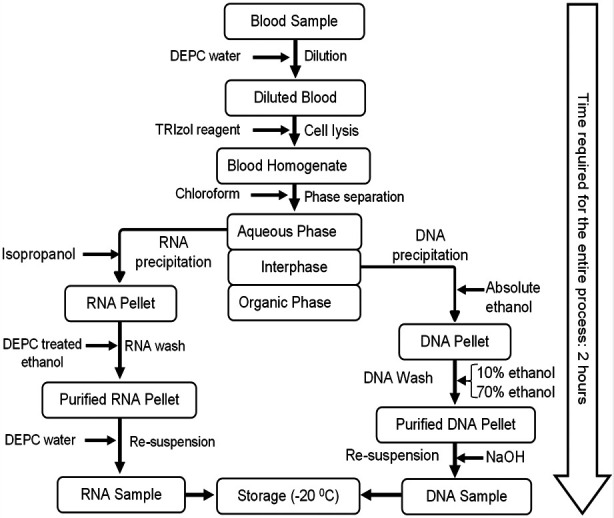
A flowchart of RNA and DNA Co-Extraction

### PCR Amplification

To assess the DNA quality, integrity and suitability for the subsequent experiment(s) as well as the effect of storage at -20°C, PCR reaction was performed with ten DNA samples after 10 weeks of extraction to amplify thrombomodulin (*THBD*) gene (124 bp) through an already established protocol.^15^ The amplified product was analyzed on 2% agarose gel, and the bands appearance was observed under UV radiation.^16^,^17^

### Data Analysis

Computer digital image analysis was done by the ImageJ software. The quality and the quantity of DNA extracted by the optimized Trizol method were compared with an existing Phenol-Chloroform method, using Student’s T-test. IBM SPSS statistics V23.0 was used to conduct the analysis. The statistical significance was set at P<0.05.

## RESULTS

**Fig.2:**
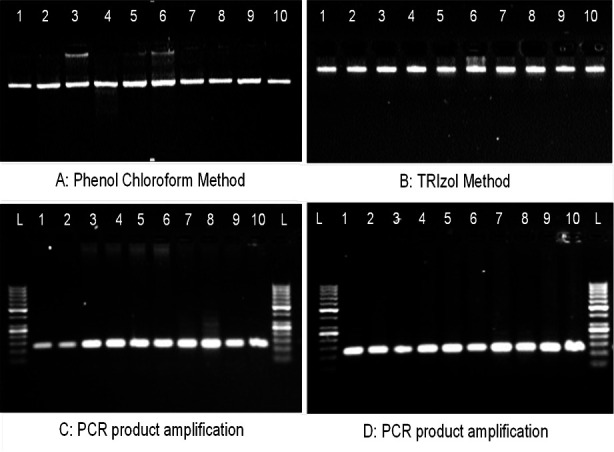
Agarose gel showing intact genomic DNA extracted from the blood of CAD patients by (A) traditional phenol-chloroform method, (B modified TRIzol™ method. (C) PCR product amplification of the extracted DNA by phenol-chloroform method, (D) PCR product amplification of the extracted DNA by modification TRIzol™ method. The PCR was run with 50 bp DNA ladder, 10 weeks after the DNA extraction to amplify THBD gene (124bp)

DNA and RNA were extracted concomitantly from ten CAD patients using both methods (TRIzol and phenol-chloroform). Results of RNA extraction using TRIzol™ method has been previously reported.^7^ DNA extraction results using the conventional phenol-chloroform method and the comparison of two results are presented in Table-I. It was observed that the quantity of DNA extracted from the TRIzol method was significantly (P<0.05) higher than phenol-chloroform method by 3-folds. However, the purity of the extracted DNA (determined by the value of A_260_:A_280_) is significantly (P<0.05) higher in the phenol-chloroform method. Moreover, the appearance of the extracted DNA on agarose gel showed intact genomic DNA, thus good integrity in both the phenol-chloroform method and TRIzol™ method (Fig.2A & B). Additionally, the PCR product amplification of TRIzol™ method revealed good quality and efficient extracted DNA suitable for downstream analysis (Fig. 2C & D). The relative percentage difference obtained from peaks of gel band densities is 0.77-1.74 (supplementary Table-I).

**Table I:** Comparison of quantity and purity of DNA extracted by methods A & B from the blood of CAD patients.

Sample ID	Method A		Method B		

	DNA Yield (ng/µL)	A_260_:A_280_	DNA Yield (ng/µL)	A_260_:A_280_	
CAD_1	59.92	2.21	122.41	1.73	
CAD_2	73.52	2.31	113.98	1.87	
CAD_3	88.16	2.50	558.90	1.62	
CAD_4	88.48	1.84	148.74	1.80	
CAD_5	85.68	2.01	433.00	1.70	
CAD_6	139.80	1.97	455.03	1.52	
CAD_7	103.00	1.90	462.21	1.98	
CAD_8	94.24	1.90	190.94	1.58	
CAD_9	200.01	1.98	241.23	1.62	
CAD_10	111.20	2.06	453.62	1.800	

*Metdod*	*Duration*	*Average A_260_:A_280_*	*A_260_:A_280_ range*	*Average DNA Yield (ng/µL)*	*Range of DNA Yield (ng/µL)*

Phenol-Chloroform Method	2 days	2.068^a^	1.84 - 2.50	104.40^a^	59.92 - 200.01
TRIzol Method	2 hours	1.72^b^	1.52 - 1.98	318.01^b^	113.98 - 558.90

Method A: phenol-chloroform method, Method B: TRIzol™ method, A_260_: Absorbance at 260nm, A_280_: Absorbance at 280nm. ^a,b^ significant difference (P<0.05).

## DISCUSSION

The concentrations of DNA or RNA was determined by the measurement of sample absorbance at 260nm. The absorbance ratio of 260/280 was used to determine nucleic acid purity or presence of contaminants in the extracted DNA or RNA samples.^18^,^19^ Our results showed that the TRIzol™ method had good DNA yield and integrity. The high concentration of DNA observed in the present TRIzol™ method compared to the phenol-chloroform method could be due to proteinase K and phenol-chloroform used in the organic method of extraction.^20^ Several experiments have reported low concentration, but good purity of DNA extracted from the phenol-chloroform method.^21^,^22^ These organic chemicals are excluded in our optimized TRIzol™ method, hence more quantity of DNA was obtained. It was also noticed that the integrity of the DNA remains uncompromised after 10 weeks of extraction. Thus, the extracted molecule can be stored at -20°C for a longer period of time. The efficiency of the nucleic acid extraction method is considered the best if the quantity, purity and integrity of extracted DNA or RNA molecule are good enough for the subsequent experiments. The result of PCR product after the amplification of *THBD* gene from extracted DNA is the evidence that this method is suitable for the subsequent experiments requiring DNA isolates.^23^

Time and cost-effectiveness are also major concerns about scientific investigations especially in limited resources setups of developing countries. In the present work, simultaneous extraction of RNA and DNA was achieved in just two hours. It is useful especially when the researcher is dealing with large sample size or when the method is extended to clinical diagnosis, where urgent laboratory reports are requested, and the patients is desperate to know his/her status. Our newly designed method is advantageous over most of the traditional methods for DNA extractions. Genomic DNA extraction from whole blood is time-consuming, as overnight incubation with proteinase K to lyse the blood cells is necessary, followed by at least two rounds of phenol-chloroform extractions.^22^ Sample treatment with proteinase K and phenol-chloroform is not only time-consuming but also a relatively expensive process. Besides, there is also an increased exposure to harmful organic chemicals that may pose health challenges to the researcher.^23^ The lower cost of extracting DNA with the modified TRIzol™ method is far cheaper than the phenol-chloroform method, enzymatic or kits method, and could make DNA extraction possible even in the laboratories with limited resources for molecular biology experiments.

The volume of samples and repetitive sample collection from the same patients are subjects of concern for both patient and the researcher. In TRIzol™ method, 0.3 ml of a sample is required to extract both the RNA and DNA, instead of 0.6 ml, if the two extraction procedures are processed separately. The method also reduces the chances of persistent neuropathic pains, complex regional pain syndrome, double sticking, anxiety and fainting resulted from venipuncture during sample collection.^24^,^25^ Similarly, the volume of blood required to obtain RNA and DNA samples will be reduced. These are advantageous especially to patients with low blood count-related diseases such as sickle cell disease, thalassemia, leukemia, aplastic anemia, iron and vitamin deficiency, as well as hemophobic subjects.

### Limitation of the study

The present study was limited to the extraction of RNA and DNA in whole blood samples. The suitability of the method for the extraction of nucleic acids on other body fluids such as plasma, saliva, breast milk, bile, cerebrospinal fluid, semen, urine, etc was not tested. Similarly, the efficiency of the method was not studied on tissue homogenates, cell lines, microorganisms, and plant material. These could be done in future.

## CONCLUSION

We presented TRIzol™ method for DNA extraction that provides a high yield of DNA molecules from blood samples of CAD patients with relatively good purity and integrity. Most importantly, extracted DNA can be successfully used to perform PCR, thus the suitability of the method to subsequent experiments was ascertained. This method is relatively cost-effective and less time-consuming, as well as minimizes the researcher exposure to toxic organic chemicals. The TRIzol™ method for RNA and DNA co-extraction is a recommended fast, simple and more economical technique. Thus, it can be adopted for routine molecular biology analyses.

### Authors’ Contributions:

All authors have accepted responsibility for the entire content of this manuscript and approved its submission.

**AD:** Was involved in conceptualization, methodology, investigation, analysis and writing original draft. **MR:** Contributed to methodology, investigation and analysis. **AL and MJK:** Contributed to conceptualization, validation, resources, project administration and supervision.
